# Growth charts for patients with Sanfilippo syndrome (Mucopolysaccharidosis type III)

**DOI:** 10.1186/s13023-019-1065-x

**Published:** 2019-05-02

**Authors:** Nicole M. Muschol, Daniel Pape, Kai Kossow, Kurt Ullrich, Laila Arash-Kaps, Julia B. Hennermann, Ralf Stücker, Sandra R. Breyer

**Affiliations:** 1 Department of Pediatrics, University Medical Center Hamburg-Eppendorf, Martinistr. 52, 20246 Hamburg, Germany; 2 Department of Medical Psychology, University Medical Center Hamburg-Eppendorf, Martinistr. 52, 20246 Hamburg, Germany; 3 Martin Zeitz Center for Rare Diseases, University Medical Center Hamburg-Eppendorf, Martinistr.52, 20246 Hamburg, Germany; 4 Villa Metabolica, Department of Pediatric and Adolescent Medicine, University Medical Center Mainz, Langenbeckstr. 1, 55131 Mainz, Germany; 5 Department of Pediatric Orthopedics, Altonaer Children’s Hospital, Bleickenallee 38, 22763 Hamburg, Germany; 6 Department of Orthopedics, University Medical Center Hamburg-Eppendorf, Martinistr. 52, 20246 Hamburg, Germany

**Keywords:** Mucopolysaccharidosis type III, Sanfilippo syndrome, Growth charts, Weight, Body height, Head circumference, Growth velocity, Body mass index, Puberty

## Abstract

**Background:**

Mucopolysaccharidosis (MPS) type III (Sanfilippo syndrome) comprises a group of rare, lysosomal storage diseases caused by the deficiency of one of four enzymes involved in the degradation of heparan sulfate. The clinical hallmark of the disease is severe neurological deterioration leading to dementia and death in the second decade of life. Adult MPS patients are generally of short stature. To date there is no clear description of the physical development of MPS III patients. The aim of this study was to document growth reference data for MPS III patients. We collected growth data of 182 German MPS III patients and were able to develop growth charts for this cohort. Growth curves for height, weight, head circumference, and body mass index were calculated and compared to German reference charts.

**Results:**

Birth height, weight and head circumference were within the physiological ranges. Both genders were significantly taller than healthy children at 2 years of age, while only male patients were taller at the age of four. Growth velocity decelerated after the ages of 4.5 and 5 years for female and male patients, respectively. Both genders were significantly shorter than the reference group at the age of 17.5 years. Head circumference was larger compared to healthy matched controls within the first 2 years of life and remained enlarged until physical maturity.

**Conclusion:**

MPS III is a not yet treatable severe neuro-degenerative disease, developing new therapeutic strategies might change the course of the disease significantly. The present charts contribute to the understanding of the natural history of MPS III. Specific growth charts represent an important tool for families and physicians as the expected height at physical maturity can be estimated and therapeutic effects can be monitored.

**Electronic supplementary material:**

The online version of this article (10.1186/s13023-019-1065-x) contains supplementary material, which is available to authorized users.

## Background

Mucopolysaccharidosis type III (Sanfilippo syndrome, MPS III) comprises a group of clinically indistinguishable, rare, autosomal recessive lysosomal storage diseases caused by the deficiency of one of four enzymes (defining the subtypes A-D) involved in the degradation of heparan sulfate (HS): heparan N-sulfatase (sulfamidase), α-N-acetylglucosaminidase (NAGLU), acetyl-coenzyme A α-glucosaminide-N-acetyltransferase and N-acetylglucosamine-6-sulfatase [1]. The incidence of MPS III in Germany has been estimated to be 1 in 63,700 births [2]. MPS IIIA is the most common subtype in Northern Europe [3]. The clinical manifestations and disease progression of the different MPS III subtypes are variable due to variations in residual enzyme activities caused by different mutations in the four affected genes [4–11]. In a former study we reported on a slowly progressive phenotype of MPS IIIA patients carrying the missense mutation p.Ser298Pro(c.892 T > C) on one allele of the *SGSH* gene [12]. Patients with MPS III have been reported to be normal at birth. In early childhood behavioral abnormalities, sleep disturbances and a delayed speech development become apparent followed by deterioration of neurological and motor function [13]. In addition, coarse facial features, hepatosplenomegaly and skeletal dysostosis multiplex are common [1, 14, 15]. Adult MPS patients are generally of short stature [16]. There are inconclusive data on physical development in MPS III patients varying from normal growth development to growth retardation [17, 18]. Recently published growth charts of Dutch MPS III patients show birth height to be normal, but significantly stunted growth from 6 years of age onwards [19].

Disease-specific growth charts are important tools for tracking growth and recognizing deviations from normal. Physicians are able to counsel parents with regard to growth expectations. Even in a patient group where growth disturbance is not the severest pathology, growth charts might be important. Impact of possible new therapies for MPS III patients could change pathological growth to normal. Therefore, descriptions of the natural history of growth in MPS III patients are important to be able to assess changes.

The aim of this study was to document growth reference data for MPS III patients and to describe the natural history of height, weight and head circumference in addition to growth spurts and clinical signs of puberty. We collected data of 182 German MPS III patients and developed charts for height, weight, head circumference and the body mass index.

## Methods

### Study population

A retrospective chart review of 195 MPS III patients from two German specialized centers, was performed. Growth data of MPS III patients previously published by our workgroup were incorporated into the study [13]. The diagnosis of MPS III was confirmed by enzymatic testing in all but two patients, in whom the diagnosis was confirmed by urinary heparan sulfate detection. We had a dropout of 11 patients. Two patients of Pakistani origin presented with extreme growth retardation and were therefore excluded. Two patients (1 female, 1 male) developed a precocious puberty. Their data were only included up to their 6th birthday, before the start of puberty and medication. The distribution of geographic ancestry was 76.4% German. In 11.5% the origin was unknown, 4.9% Turkish, 2.2% Sinti and Romanies, 1.1% Spanish, 1.1% Italian, 0.5% Moroccan, 0.5% Polish, 0.5% Saudi-Arabian, 0.5% Swiss and 0.5% Syrian. We included 16 premature born patients (27.-36. week of gestation). Data from patients born before 35th week of gestation (*n* = 7) were adjusted to the calculated date of birth.

Twenty-five boys and 24 girls had already died at the time of data collection. The mean age at death was 16.8 years (SD 4.88, range 8.3–27.5 years) for boys and 21.2 years (SD 9.97, range 10.3–41.8 years) for girls. Overall 182 patients (96 male and 86 female) were included in the study. These patients had a mean age of 17.7 years (SD 7.19, range 4.3–37.5 years) in males and 19.1 year (SD 8.86, range 3.8–41.8 years) in females at the time of assessment. Divided into subtypes, we had 135 MPS IIIA, 34 MPS IIIB and 11 MPS IIIC patients. Two patients had an unclassified subtype. Genetic data could be collected in 69 MPS IIIA patients. In this group we had 10 patients (3 male, 7 female) carrying the mutation p.Ser298Pro on one allele of the *SGSH* gene.

Data from 1967 until 2015 were included. Gender, height, weight and head circumference (HC) were analyzed from birth until 21 years of age. Body mass index (BMI) was calculated from these data. Furthermore, data for age at thelarche, pubarche and menarche in girls and pubarche and growth of beard for boys were collected. Signs of puberty were retrospectively assessed by parent interviews. Patients over the age of 18 years were assumed to be fully grown. Data after the age of 18 years were integrated in the measurements for 18 year old patients.

Our data were compared with the German KiGGS (Kinder- und Jugendgesundheits-Survey) reference percentiles for anthropometric measurements [20]. As the KiGGS percentiles included a migrant percentage of 17%, patients with migrant background were not analyzed separately.

### Statistical analysis

The statistical analysis was performed by using SPSS 20.0 for Windows, 22.0 for Macintosh (SPSS inc., Chicago, IL, USA) and Microsoft Excel 2010 for Windows (Microsoft Corporation, Redmond, WA, USA). Mean height, weight, HC, and body mass index (BMI) were compared with a healthy German reference population [20] using one-sample t test. For independent groups, one-way ANOVA to verify the hypothesis of equality of means, was used. For nonparametric data, the statistical test of independence based on chi-square was analyzed. A *p* value < 0.05 was considered statistically significant.

Growth curves for height, weight, head circumference, and BMI were calculated and plotted for each gender using the package gamlss (Version 4.3.3) [21] in R (Version 3.5.0) [22], which uses the lambda *λ* (power in the Box-Cox transformation), mu *μ* (Median) and sigma *σ* (the generalized coefficient of variation) LMS method [23]. The Q test was conducted to evaluate the model fit [24]. With the assumption that the residuals follow a normal distribution and given LMS parameters, a smoothed distribution of an anthropometric variable can be calculated [25]. The calculated LMS parameters were converted back to Excel to create charts including the German growth charts of 2013 [20, 26]. We were not able to see a statistically significant secular growth change [27, 28], nor secular trend for weight, HC and BMI in patients born before and after 1990. Therefore, we did not perform a transformation of this data.

## Results

### Height

**Fig. 1:**
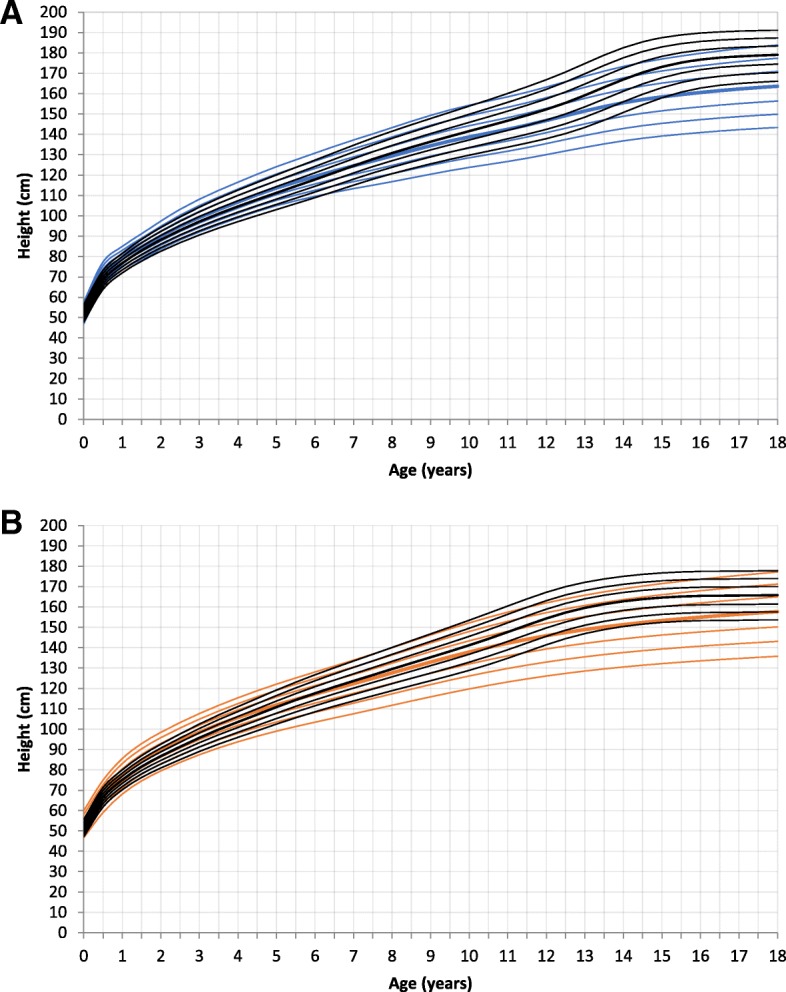
Constructed Growth charts for MPS III patients in relation to the KiGGS reference charts (black); **a** for boys (blue); **b** for girls (orange)

The mean of longitudinal measurements per patient was 7.6 (Standard deviation (SD) 4.64). The mean birth height was 52.3 cm (SD 2.88) for male (*n* = 76) and 51.1 cm (SD 2.8) for female newborns (*n* = 75). There was no significant difference to the reference group. At the age of 2 and 4 years male MPS III patients were significantly taller than the normal population (age 2 years: mean 90.1 cm, SD 3.62, *p* = 0.001, mean reference group 88.2 cm; age 4 years: mean 106.6 cm, SD 4.39, *p* = 0.008, mean reference group 104.6 cm). Female patients were significantly taller than the reference group at 2 years of age (mean 88.3 cm, SD 4.38, *p* = 0.017, mean reference group 86.7 cm), but there was no significant difference at 4 years of age (104.5 cm versus 103.5 cm). Due to the limited availability of measurements between the ages of 5–17 years we were not able to obtain a statistically robust dataset for the comparison of height with the reference points. Descriptively, patients were shorter at age 5–7 years compared to the reference group. At 17.5 years of age both genders were significantly shorter than the reference group (male: mean 163.1 cm, SD 11.90, *p* < 0.000, mean reference group 178.7 cm; female: mean 155.9 cm, SD 11.33, *p* = 0.001, mean reference group 165.7 cm) (Fig. 1 and Additional file 1: Figure RD1). Analyzing the subtypes, at the ages of 2 and 4 years, MPS IIIC patients were the tallest subgroup (age 2: mean 92.2 cm, SD 2.49; age 4: mean 111.5 cm, SD 2.12), followed by MPS IIIA (age 2: mean 89.3 cm, SD 4.14; age 4: mean 105.8 cm, SD 5.14). MPS IIIB patients were the shortest ones (age 2: mean 88.2 cm, SD 4.08; age 4: mean 103.7 cm, SD 6.12). However, this is only a tendency, as the differences between the MPS groups were not significant, due to the small number of cases.

The genetically expected height at maturity was calculated for 31 patients as described by Tanner [29]. All patients were significantly shorter at maturity than genetically expected.

### Growth velocity

**Fig. 2:**
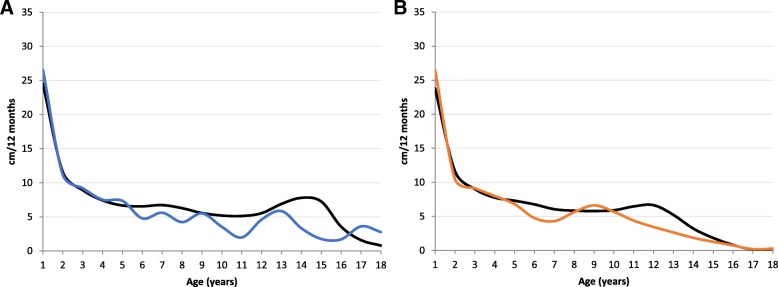
Constructed Growth Velocity (cm/per year) for MPS III patients versus KiGGS (black line), **a** for boys (blue); **b** for girls (orange)

Male and female MPS III patients showed a higher growth rate in the first year of life compared to the reference population. They remained parallel to the reference charts during the following years and decelerate after 4.5 years and 5 years for female and male patients, respectively. Male patients showed 5 growth spurts with accelerated peaks at 5, 7, 9, 13 and 17 years of age. All peaks, except the first and last one, were reduced in velocity compared to the reference group. Female patients had only one growth spurt with 9 years of age. The growth velocity was higher compared to the reference group at this age but decreased rapidly and continuously (Fig. 2).

### Clinical signs of puberty

Data for age at thelarche, pubarche, and menarche as well as growth of beard for male patients were collected. Documentation of the status of the menarche was available in 45/86 female patients. Mean age at menarche was 13.3 years (SD 2.41, range 9.5–21 years). Data of thelarche was documented in 25/86 patients. Thelarche occurred at a mean age of 12.6 years (SD 2.18, range 9.8–16 years). Signs of pubarche were documented in 51/96 male patients with a mean age of 12.6 years (SD 2.75, range 7.0–18.0 years) and in 49/86 female patients with a mean of 13.9 years (SD 3.50, range 9.0–25.0 years). Beard growth was seen in 17/33 patients (mean 14.7 years, SD 3.07, range 9.0–20.0 years). Excluded from analysis were two MPS IIIA patients (1 male, 1 female) with central precocious puberty. The female patient had her pubarche at 6.75 and menarche at 6.5 years of age. Thelarche was seen with 7.5 years. The male patient was 7 years old at pubarche. Both patients were treated with gonatotropin-releasing hormone agonists.

### Weight and body mass index

**Fig. 3:**
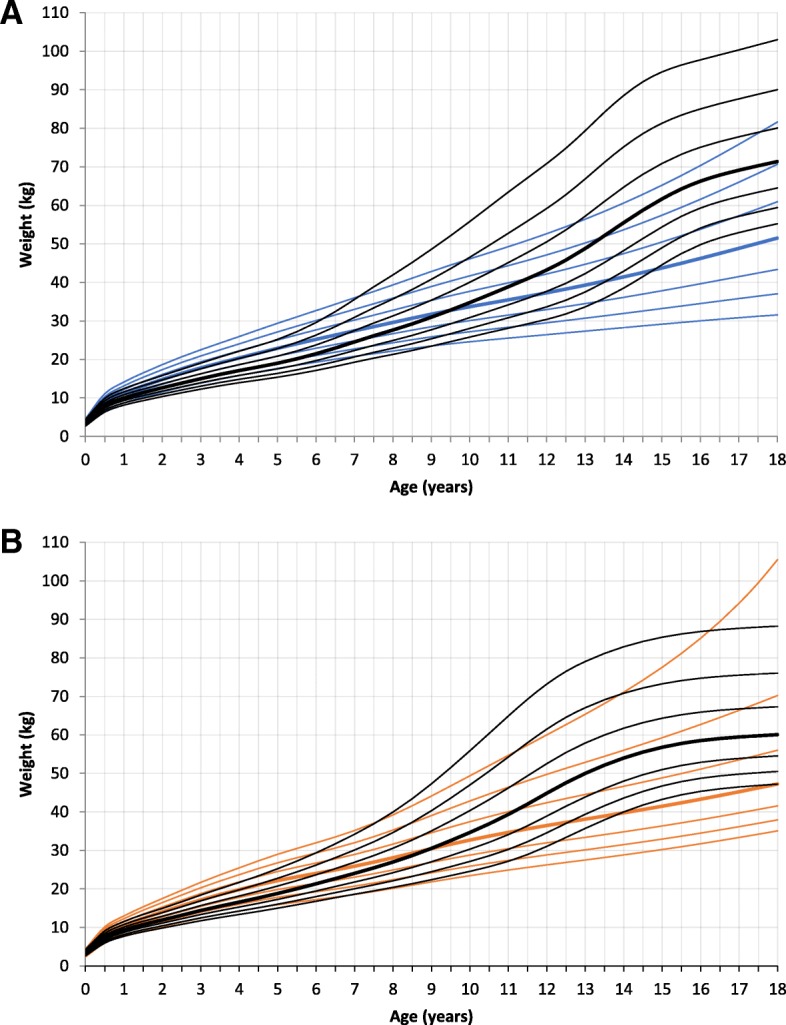
Constructed charts for weight (kg) for MPS III patients in relation to the KiGGS reference charts (black); **a** for boys (blue); **b** for girls (orange)

MPS III patients had the same birth weight as the reference group. The mean weight at birth was 3.5 kg (SD 0.6) for male patients and 3.4 kg (SD 0.59) for female patients. At age 2 and 4 both genders had a significantly higher weight compared to the reference group. Weight for male patients at age 4 was in mean 20.0 kg (SD 2.84, *p* < 0.000, mean reference group: 17.2 kg) and for female patients 18.7 kg (SD 3.01, *p* = 0.001, mean reference group: 16.6 kg). At age 18 male and female patients were significantly lighter (male: mean 52.9 kg, SD 15.74, *p* > 0.000, mean reference group: 71.4 kg; female: mean 49.8 kg, SD 9.78, *p* < 0.000, mean reference group: 60.1 kg) (Fig. 3 and Additional file 2: Figure RD2). There was no difference between the subgroups A-C.

**Fig. 4:**
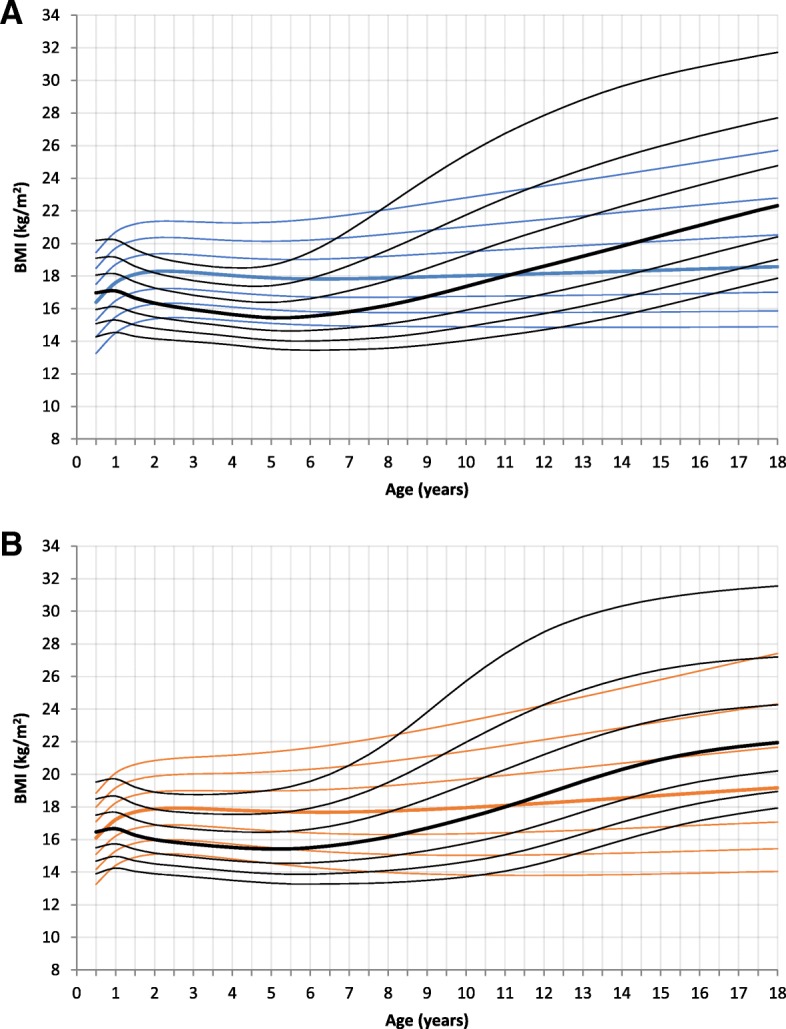
Constructed charts for the BMI (kg/m^2^) for MPS III patients in relation to the KiGGS reference charts (black); **a** for boys (blue); **b** for girls (orange)

BMI data was not available for newborns in the reference charts. BMI was significantly higher for 2 and 4-year-old patients of both genders. The mean BMI off male patients was 17.7 kg/m^2^ (SD 1.6, p < 0.000, mean reference group: 16 kg/m^2^) at 2 years of age and 17.0 kg/m^2^ (SD 1.49, p < 0.000, mean reference group: 15.5 kg/m^2^) at 4 years of age. The BMI of male patients at 2 years of age was 18.2 kg/m^2^ (SD 1.39, p < 0.000, mean reference group: 16.3 kg/m^2^), at 4 years of age 17.8 kg/m^2^(SD 1.6, *p* < 0.000, mean reference group: 15.6 kg/m^2^). BMI of male patients age 18 years (mean 20.1 kg/m^2^, SD 3.24, *p* = 0.014) was significantly lower compared to the reference group (mean 22.3 kg/m^2^), but the sample size was small (*n* = 17). Female patients with 18 years of age showed no statistically significant difference to the reference group, but a tendency towards a lower BMI (mean patients: 20.7 kg/m^2^, SD 2.98; mean reference group: 22 kg/m^2^) (Fig. 4 and Additional file 3: Figure RD3).

### Head circumference

**Fig. 5:**
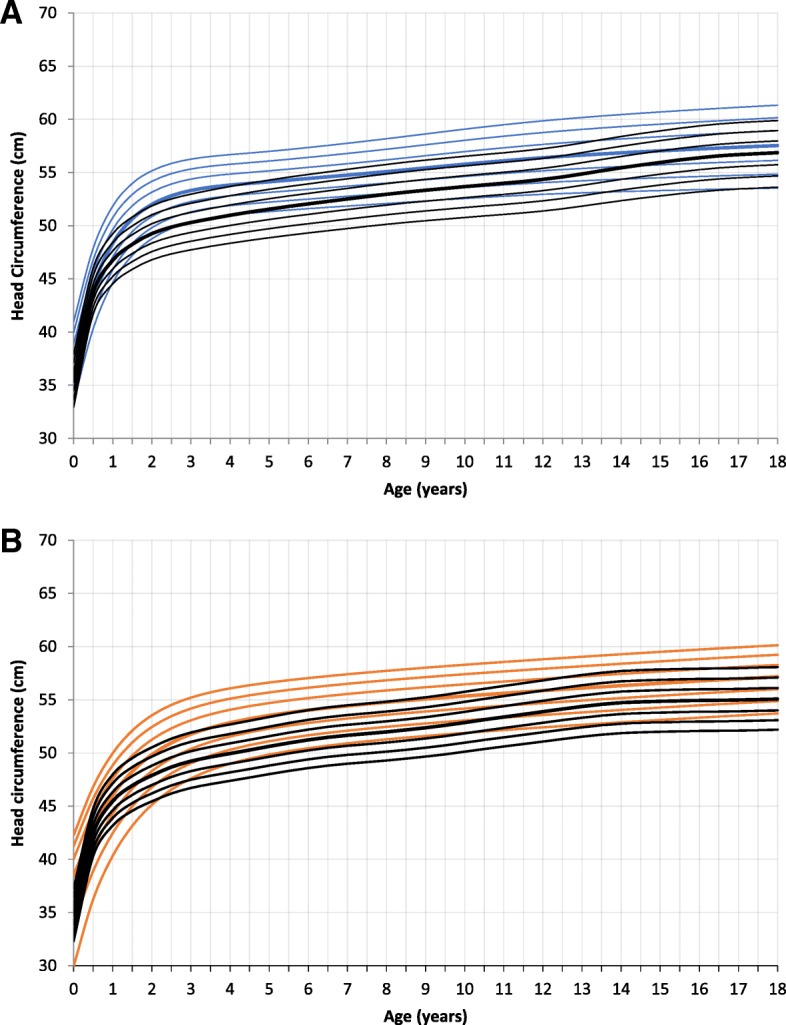
Constructed charts for the head circumference (cm) for MPS III patients in relation to the KiGGS reference charts (black); **a** for boys (blue); **b** for girls (orange)

At birth head circumference was not significantly different to the reference group for both genders. This changed after 2 years of age. MPS III patients had a significant larger HC at age 2 and 4 years of age. Two year old boys had a HC comparable to a 5 year old healthy male (mean male patient at 2 years: 51.7 cm, SD 1.52, *p* < 0.000, mean reference group 49.3 cm), girls of age 2 had a HC similar to a 4.5 year old healthy female (mean female patient at 2 years: 50.4 cm, SD 1.22, p < 0.000, mean reference group 47.9 cm). At 4 years of age the HC was still significantly larger compared to healthy controls (male: mean 53.7 cm, SD 1.6, p < 0.000, mean reference group 51 cm; female: mean 52.4 cm, SD 1.41, p < 0.000, mean reference group 49.9 cm). Adult male patients had a HC in mean of 57.9 cm (SD 1.44), females in mean of 58.6 cm (SD 1.25) (Fig. 5 and Additional file 4: Figure RD4). Due to the small sample size we were not able to calculate significances for fully grown patients.

### MPS IIIA patients with the p.Ser298Pro mutation on one allele

Genetic data were available from 69 MPS IIIA patients. In 3 male and 7 female patients the p.Ser298Pro mutation was identified on one allele. There was no significant difference in growth between male and female patients with p.Ser298Pro. In comparison to MPS IIIA patients with other genetic mutations these patients were slightly smaller at birth (mean 51 cm, SD 2.58 versus 51.8 cm, SD 2.93) and significantly smaller (mean 86.4 cm, SD 4.9, *p* = 0.022 versus 90.4 cm, SD 3.99) at 2 years of age. Two years later they were still shorter in relation to other MPS IIIA patients. The final length of patients with the p.Ser298Pro mutation was higher than in patients without this mutation (mean 166.2 cm, SD 10.4 versus mean 156.8 cm, SD 12.5). MPS IIIA patients with the p.Ser298Pro mutation were slightly lighter at birth (mean 3.3 kg, SD 0.36 versus 3.5 kg, SD 0.57) compared to MPS IIIA patients with other mutations. At age 2 und 4 they were significantly lighter (age 2: mean 12.4 kg, SD 2.07, *p* = 0.001 versus other mutations: 14.8 kg, SD 1.55; age 4: mean 17.3 kg, SD 2.76, p = 0.022 versus other mutations 20.2 kg, SD 2.51). Fully grown patients with the p.Ser298Pro mutation were significantly heavier (mean 59.9 kg, SD 11.12, *p* = 0.031 versus mean 47.7 kg, SD 7.69). Head circumference was slightly decreased from birth up to age 4, but not significantly different to patients without the mutation. We had no data for HC in mature patients carrying the p.Ser298Pro mutation.

## Discussion

This study presents growth charts for a cohort of German MPS III patients. Growth curves for height, weight, head circumference, and body mass index were calculated from birth to 18 years of age and compared to German reference charts.

Newborn height, weight and HC were not significantly different to the reference charts. At the age of 2 years this changed. Affected boys and girls in this age group demonstrated a significantly higher body height, weight, BMI, and HC. The growth pattern of MPS III patients in our study was characterized by accelerated growth speed in the first year of life, with a subsequent deceleration and reduced height at maturity. These findings are similar to recently published data of a Dutch MPS III population. De Ruijter et al. described a normal birth weight and height, but showed a significantly stunted growth from the age of 6 years onwards [19]. Adult height of Dutch MPS III patients was reduced compared to Dutch reference charts. In contrast to the German MPS III cohort, male and female Dutch MPS III patients were 6.6 cm and 9.5 cm taller. This difference between the two countries might be explained by the higher incidence of intermediate and attenuated disease in the Dutch MPS III cohort (39.8% of patients) and to a lesser extent by the general genetic background of the two populations [19, 30]. The difference between the two countries also applies to the weight. Only adult BMI of MPS III patients was similar in the two groups (female: German 20.7 kg/m^2^, Dutch 20.2 kg/m^2^, male: German 20.1 kg/m^2^, Dutch 20.4 kg/m^2^).

The p.Ser298Pro genotype is present in about 10% of alleles in German patients with MPS IIIA. Patients with p.Ser298Pro genotype showed a more physiological growth with a higher body height at maturity. This reflects the milder clinical phenotype of patients with p.Ser298Pro genotype compared to classical MPS IIIA patients [12].

Increased height at birth as well as accelerated growth in infancy has been reported for MPS I, MPS II, III, IVA, VI and VII [16]. However, all MPS types show a deceleration of growth with short stature at physical maturity [16]. This also applies to MPS III, but is less pronounced. The beginning of growth retardation is not consistent between the different MPS subtypes [16]. It is reported that boys with Hurler syndrome (MPS IH) reach a body height below the 3rd percentile after 24 months of life [31]. MPS II patients decrease in growth rate from approximately 2.5 years, dropping below the lower limit of normal at approximately 7 years of age [32]. In MPS IVA patients the mean height of both genders start to fall markedly below the − 2 SD value at 4 years of age [33].

Growth velocity has not yet been described in MPS III patients. In the present study multiple acceleration peaks were seen during growth in male MPS III patients. In contrast, female patients showed only one acceleration peak with 9 years of age. Clinical signs of puberty were not correlated to these growth spurts. Parini et al. also described a lack of pubertal growth spurt in MPS II [32]. Furthermore, Quartel et al. did not observe increased acceleration in growth during pre-teen or early-teen years in MPS VI patients [34]. Growth velocity for MPS IVA patients did not show acceleration in the first year of life, but multiple acceleration peaks during childhood and adolescence [33].

The incidence of precocious puberty was 1.1% (1 female, 1 male) in the present study, and therefore lower than reported in the literature. Concolino et al. observed precocious puberty in 2/10 MPS IIIA patients [35]. Tylki-Szymanska et al. found precocious puberty in three males in a cohort of 46 MPS IIIA patients (6.5% of the total cohort and 13% of the male patients) [36].

The pathophysiology of the short stature and the altered growth pattern in MPS III is not completely understood. Reports from the literature suggest a combination of several pathological mechanisms in bone formation, bone maturation, as well as endocrinological abnormalities [37–39]. The glycosaminoglycan HS is assumed to have a lower impact on growth retardation than dermatan sulfate (DS) or keratan sulfate (KS). However, HS is an important player in the regulation of growth [40, 41]. In addition, growth hormone/insulin-like growth factors (IGF-1) deficiency or resistance has been reported in single patients with MPS IIIA [34].

It has been hypothesized, that GAG storage triggers a complex pathogenic cascade of abnormal biological mechanisms such as disruption of the extracellular matrix [42], alteration of signal transduction pathways, modulation of cytokines and other inflammatory mediators, and alteration of the intracellular targeting pathways, endocytosis, apoptosis and autophagy [43]. In the recent past, many studies on various MPS animal models have shown early abnormalities of chondrocyte organization in the growth plate and architecture of cortical bone [19, 32, 42–48].

Limitations of this study are the retrospective design and the small number of data from 5 to 17 years of age (Additional file 5: Table S1). In the course of the disease patients become disabled and agitated which can lead to difficulties performing measurements.

## Conclusion

In conclusion, patients with MPS III show a normal weight, height and head circumference at birth. In the first year of life growth acceleration is observed. Deceleration of growth in childhood and adolescence leads to a shorter height in adulthood than genetically expected.

MPS III is a not yet treatable severe neuro-degenerative disease, developing new therapeutic strategies might change the course of the disease significantly. The present charts contribute to the understanding of the natural history of MPS III. Specific growth charts represent an important tool for families and physicians as the expected height at the end of growth can be estimated and therapeutic effects can be monitored (Additional file 6: Figure C1, Additional file 7: Figure C2, Additional file 8: Figure C3 and Additional file 9: Figure C4).

## Additional files

Additional file 1: **Figure RD1.** Raw data of height (cm), showing the construction of the charts with individual data points. (DOCX 142 kb)

Additional file 2: **Figure RD2.** Raw data of weight (kg), showing the construction of the charts with individual data points. (DOCX 145 kb)

Additional file 3: **Figure RD3.** Raw data of BMI (kg/m^2^), showing the construction of the charts with individual data points (DOCX 130 kb)

Additional file 4: **Figure RD4.** Raw data of head circumference (cm), showing the construction of the charts with individual data points (DOCX 144 kb)

Additional file 5: **Table S1.** Number of measurements for male and female patients for height, weight and head circumference in relation to the age groups (XLSX 11 kb)

Additional file 6: **Figure C1.** Reconstructed charts for growth (cm) for MPS III patients, A: for boys (blue); B: for girls (orange). (DOCX 23 kb)

Additional file 7: **Figure C2.** Reconstructed charts for weight (kg) for MPS III patients, A: for boys (blue); B: for girls (orange). (DOCX 23 kb)

Additional file 8: **Figure C3.** Reconstructed charts for BMI (kg/m^2^) for MPS III patients, A: for boys (blue); B: for girls (orange). (DOCX 79 kb)

Additional file 9: **Figure C4.** Reconstructed charts for head circumference (cm) for MPS III patients, A: for boys (blue); B: for girls (orange). (DOCX 21 kb)

## Abbreviations

BMI: Body mass index
DS: Dermatan sulfate
GAG: Glycosaminoglycan
HC: Head circumference
HS: Heparan sulfate
IGF: Insulin-like growth factor
KiGGS: Kinder- und Jugend-Gesundheits-Survey
KS: Keratan sulfate
LMS: Lambda, mu, sigma
MPS: Mucopolysaccharidosis
SD: Standard deviation
