# Supplementary figures and images for: Growth charts for patients with Sanfilippo syndrome (Mucopolysaccharidosis type III)
Source: Orphanet J Rare Dis. 2019 May 2;14:93. doi: 10.1186/s13023-019-1065-x (PMC6498678; doi:10.1186/s13023-019-1065-x)

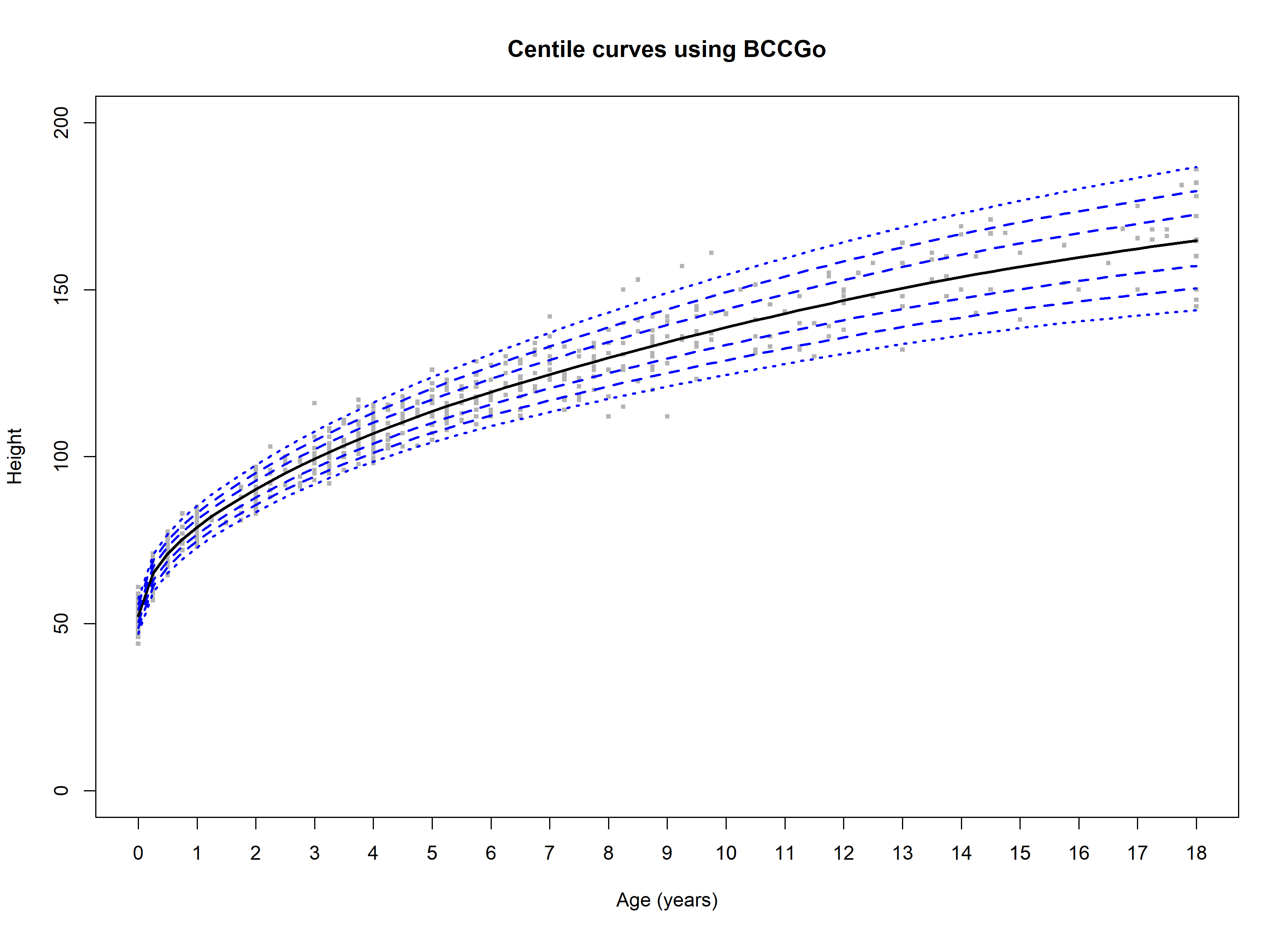


**A**


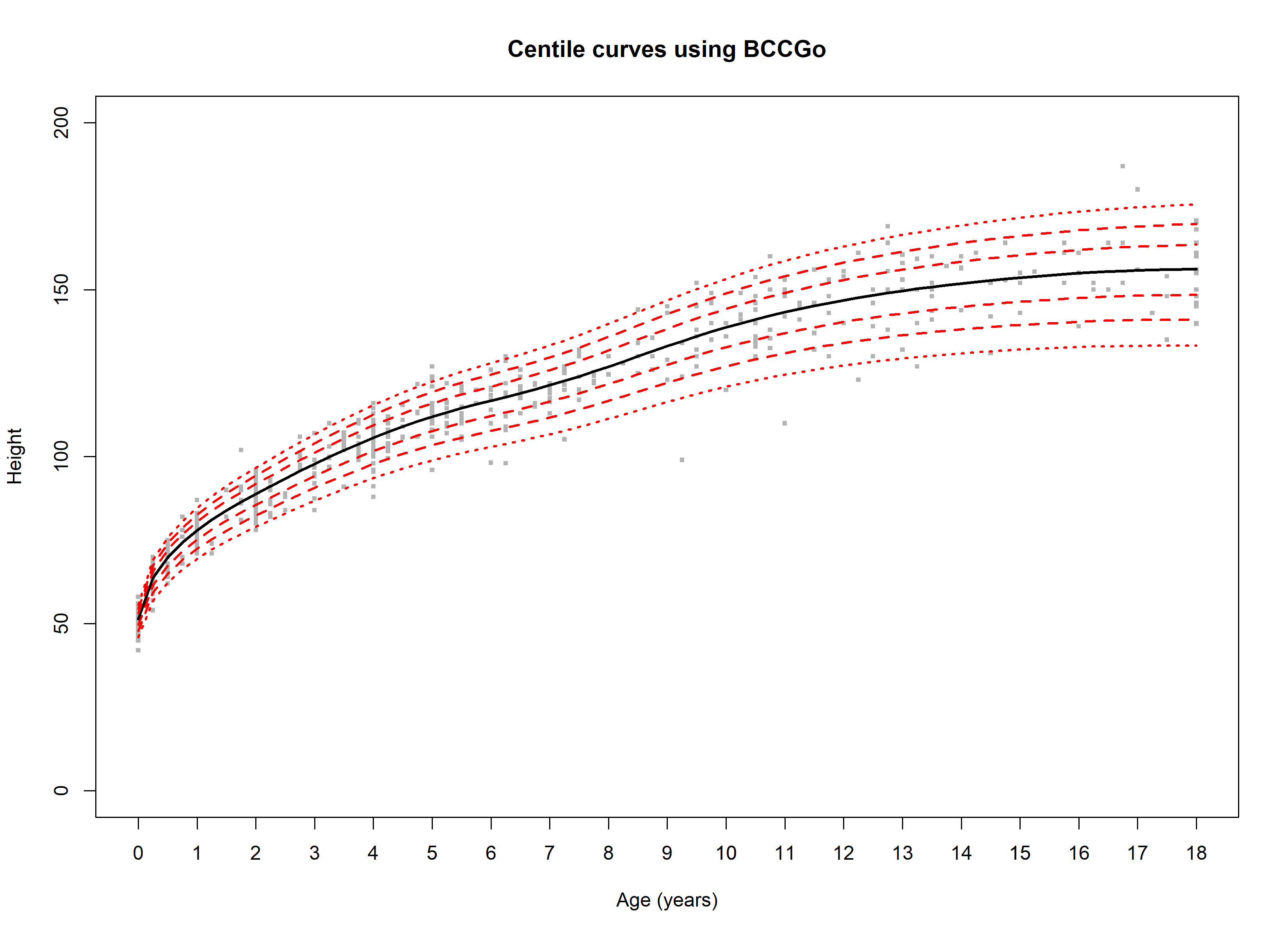


**B**

Supplement: Supplementary file 1 — Figure RD1. Raw data of height (cm), showing the construction of the charts with individual data points. (DOCX 142 kb) [file 13023_2019_1065_MOESM1_ESM.docx]

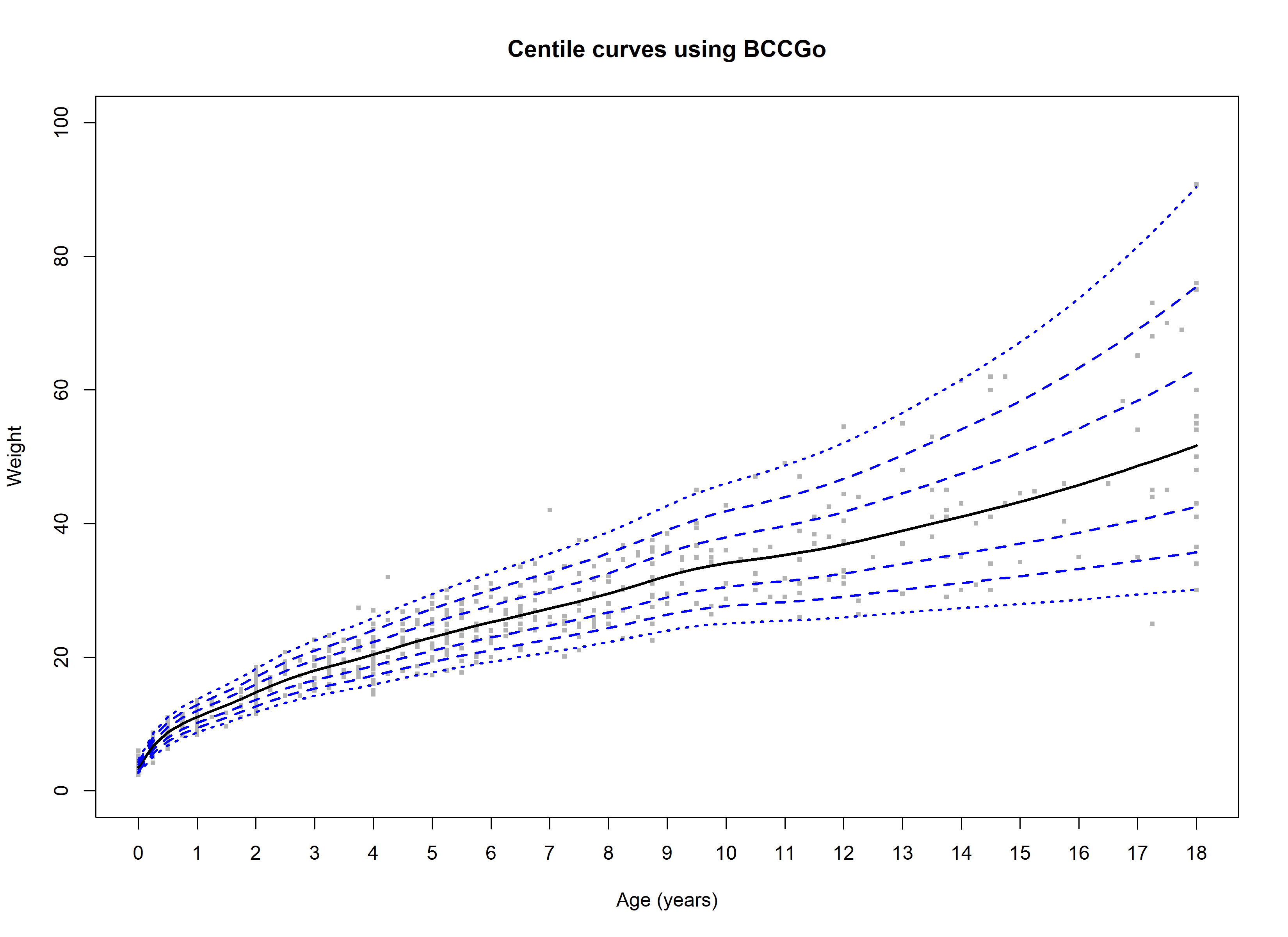


**A**


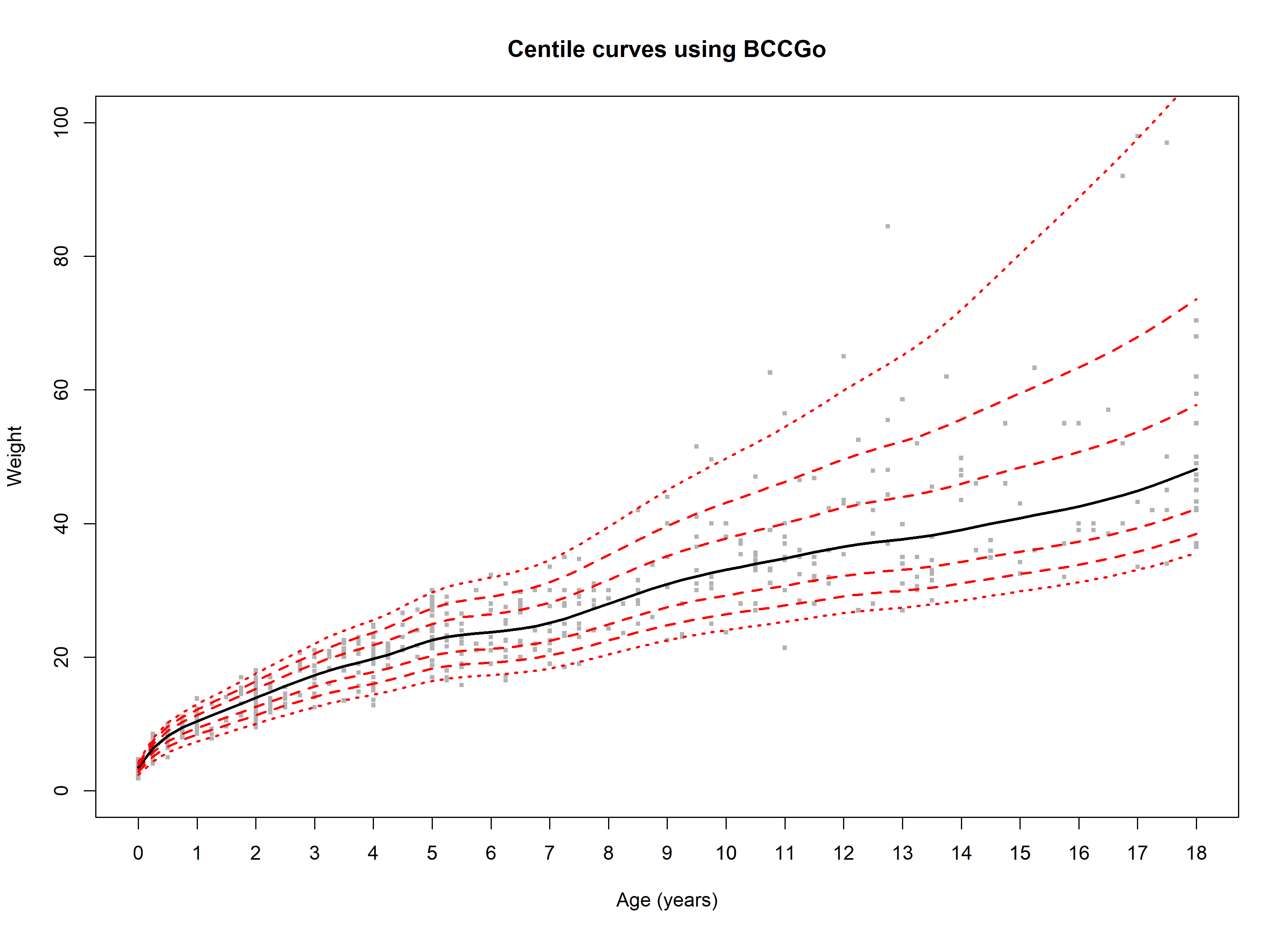


**B**

Supplement: Supplementary file 2 — Figure RD2. Raw data of weight (kg), showing the construction of the charts with individual data points. (DOCX 145 kb) [file 13023_2019_1065_MOESM2_ESM.docx]

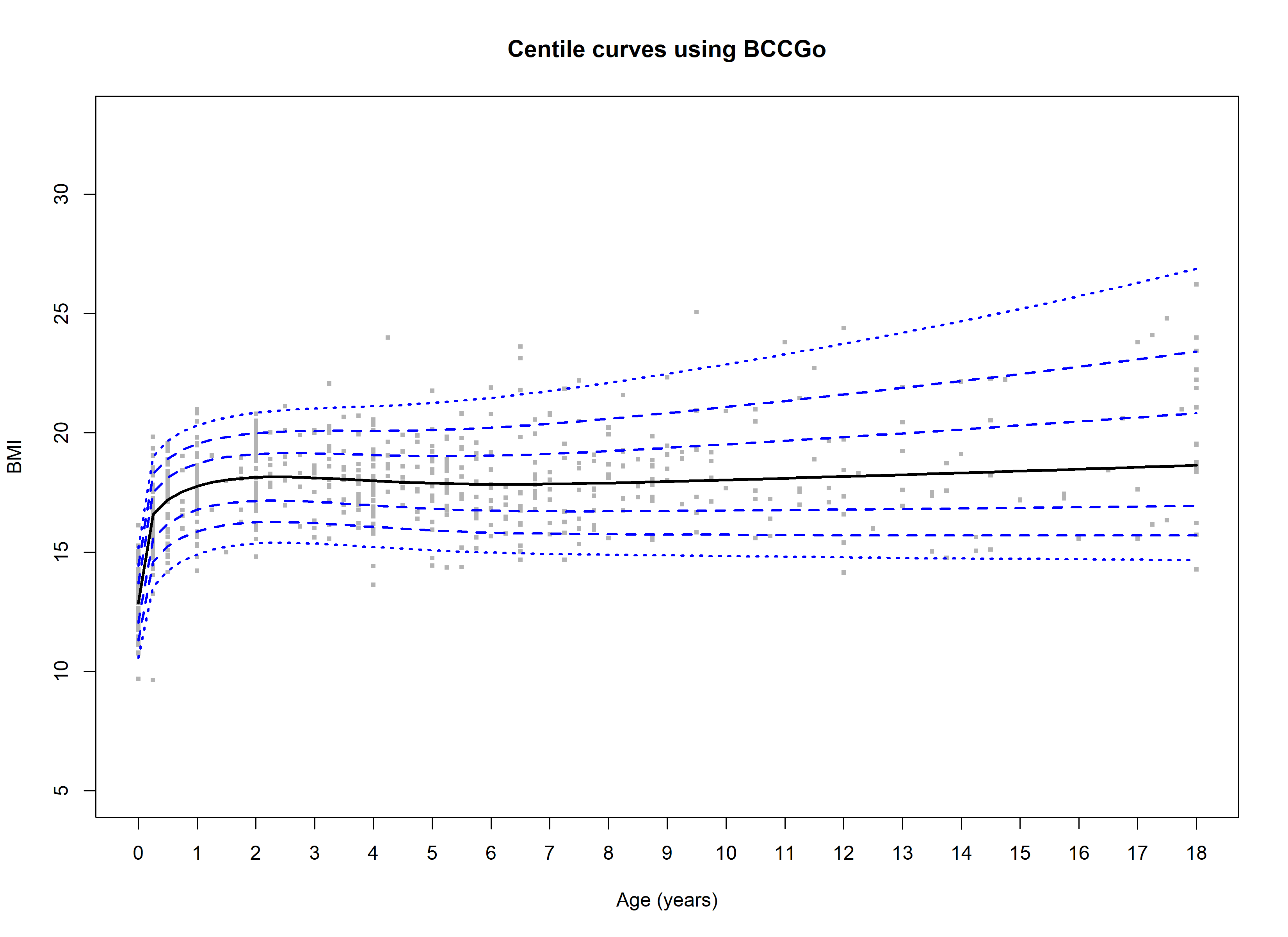


**A**


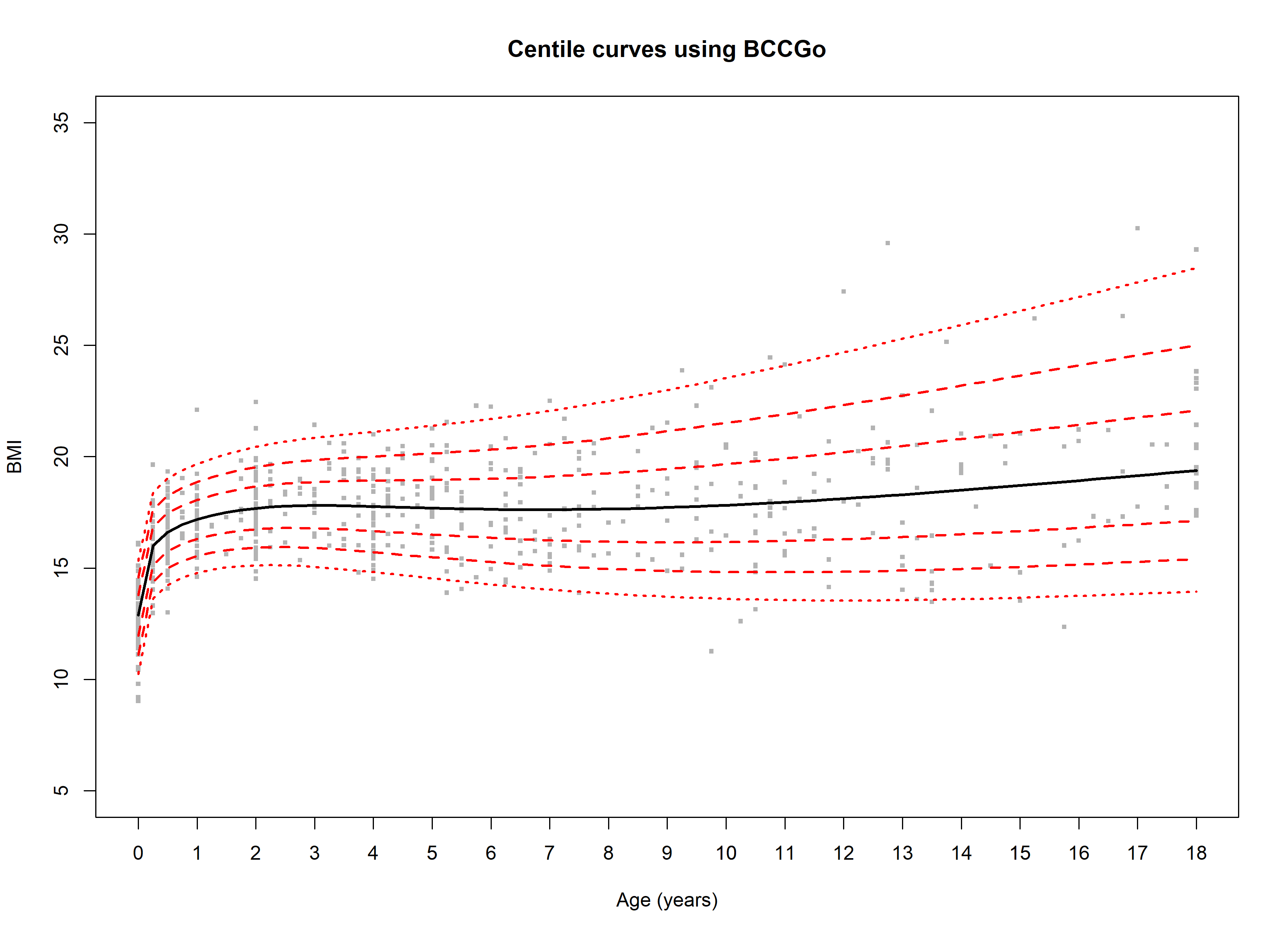


**B**

Supplement: Supplementary file 3 — Figure RD3. Raw data of BMI (kg/m2), showing the construction of the charts with individual data points (DOCX 130 kb) [file 13023_2019_1065_MOESM3_ESM.docx]

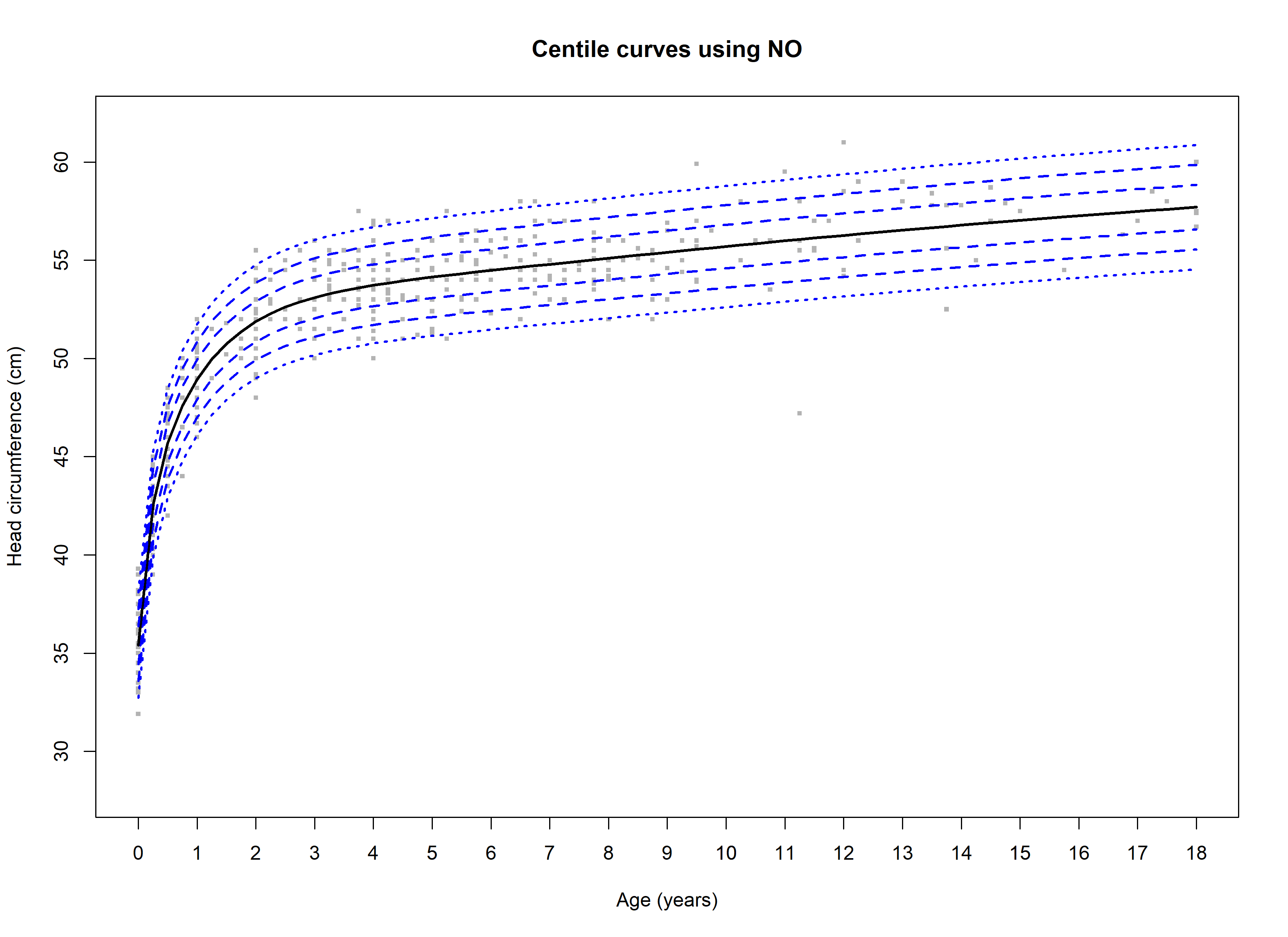


**A**


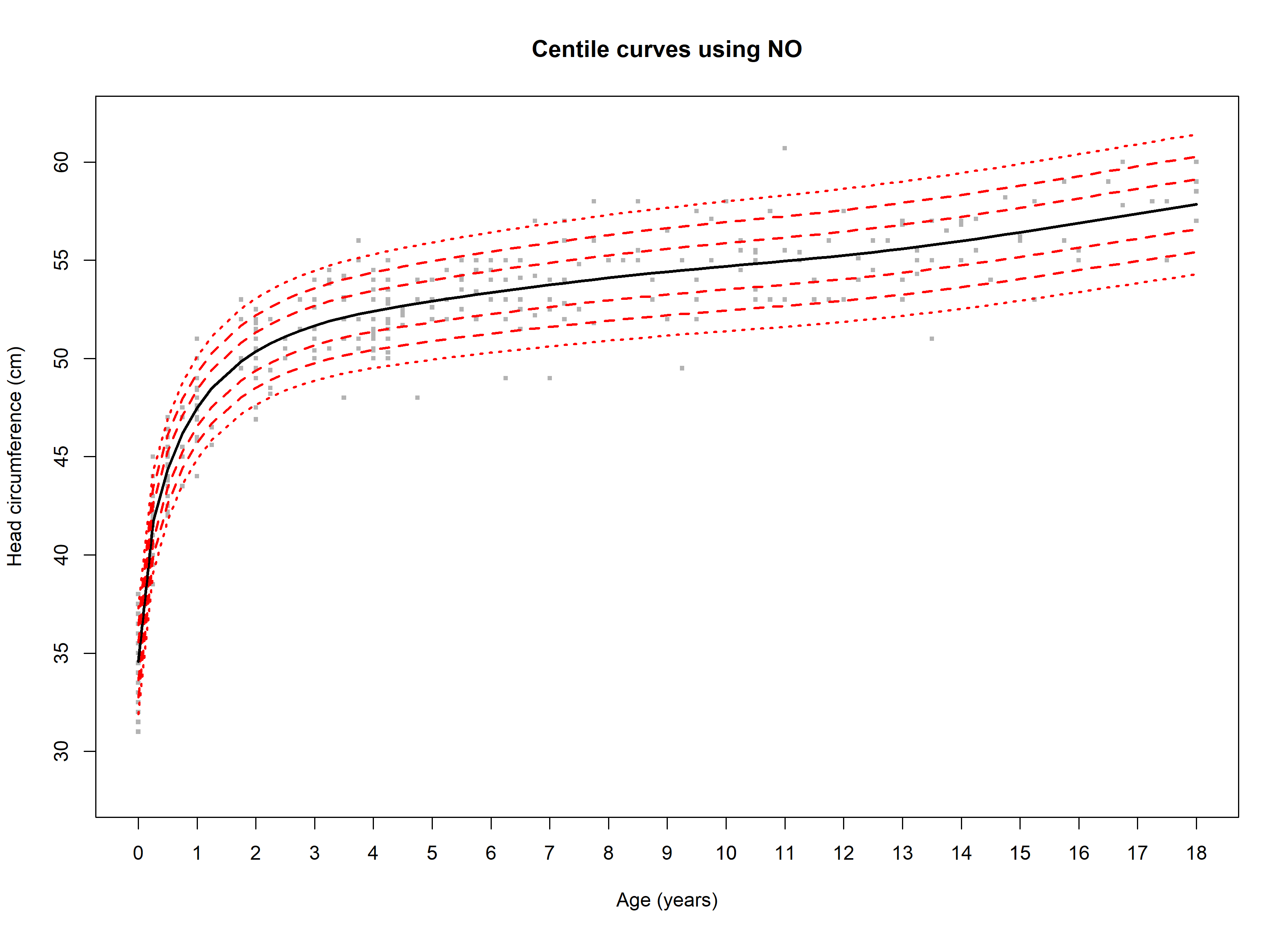


**B**

Supplement: Supplementary file 4 — Figure RD4. Raw data of head circumference (cm), showing the construction of the charts with individual data points (DOCX 144 kb) [file 13023_2019_1065_MOESM4_ESM.docx]
