# Supplementary material for: Growth charts for patients with Sanfilippo syndrome (Mucopolysaccharidosis type III)
Source: Orphanet J Rare Dis. 2019 May 2;14:93. doi: 10.1186/s13023-019-1065-x (PMC6498678; doi:10.1186/s13023-019-1065-x)
Supplement: Supplementary file 7 — Figure C2. Reconstructed charts for weight (kg) for MPS III patients, A: for boys (blue); B: for girls (orange). (DOCX 23 kb) [file 13023_2019_1065_MOESM7_ESM.docx]

**A**

**B**

**B**
