# Supplementary material for: Growth charts for patients with Sanfilippo syndrome (Mucopolysaccharidosis type III)
Source: Orphanet J Rare Dis. 2019 May 2;14:93. doi: 10.1186/s13023-019-1065-x (PMC6498678; doi:10.1186/s13023-019-1065-x)
Supplement: Supplementary file 4 [file 13023_2019_1065_MOESM8_ESM.docx]

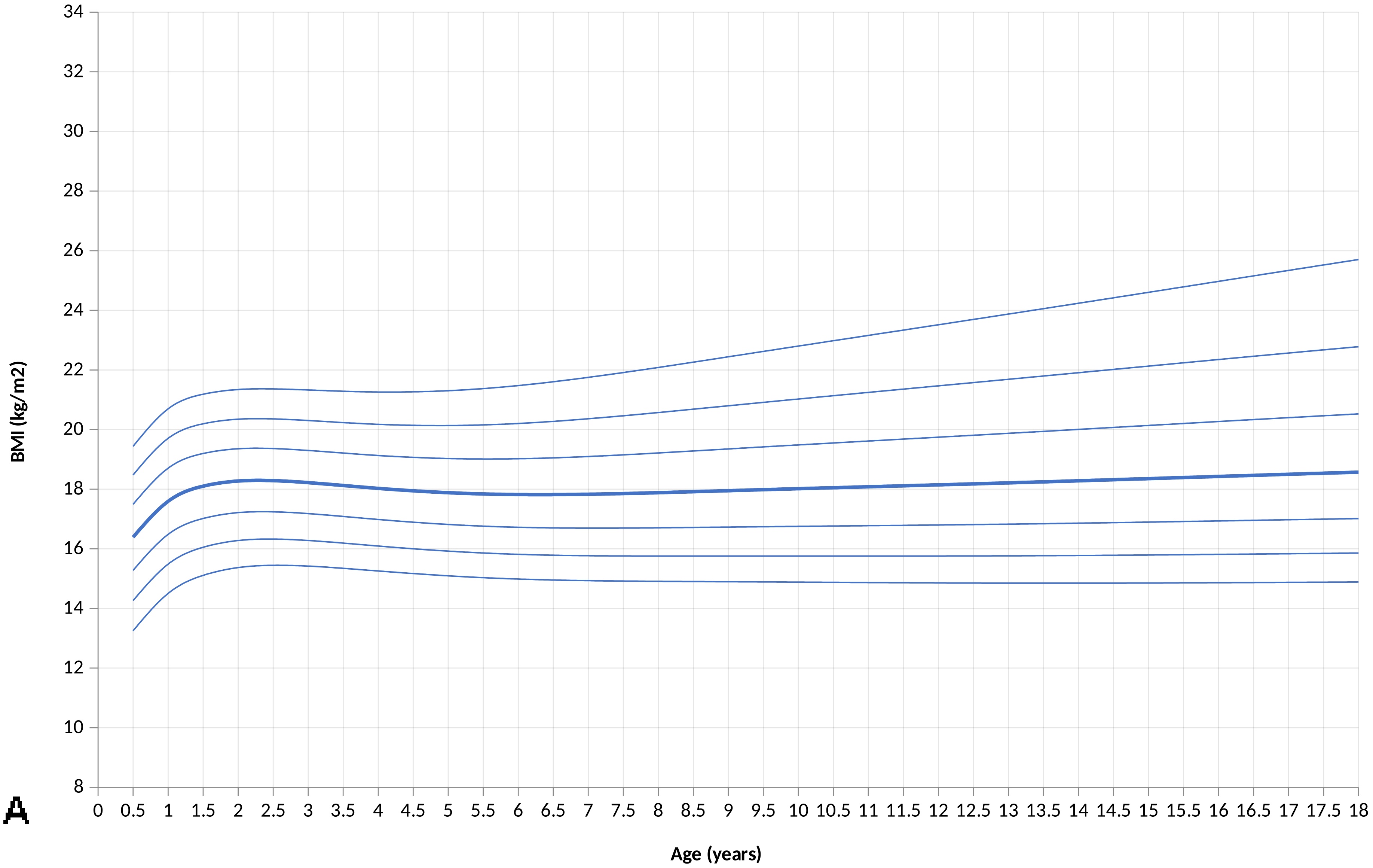

34
32
30
28
26
24
22
20
18
16
14
12
10
8
BMI (kg/m2)
0 0.5 1 1.5 2 2.5 3 3.5 4 4.5 5 5.5 6 6.5 7 7.5 8 8.5 9 9.5 10 10.5 11 11.5 12 12.5 13 13.5 14 14.5 15 15.5 16 16.5 17 17.5 18
Age (years)
A

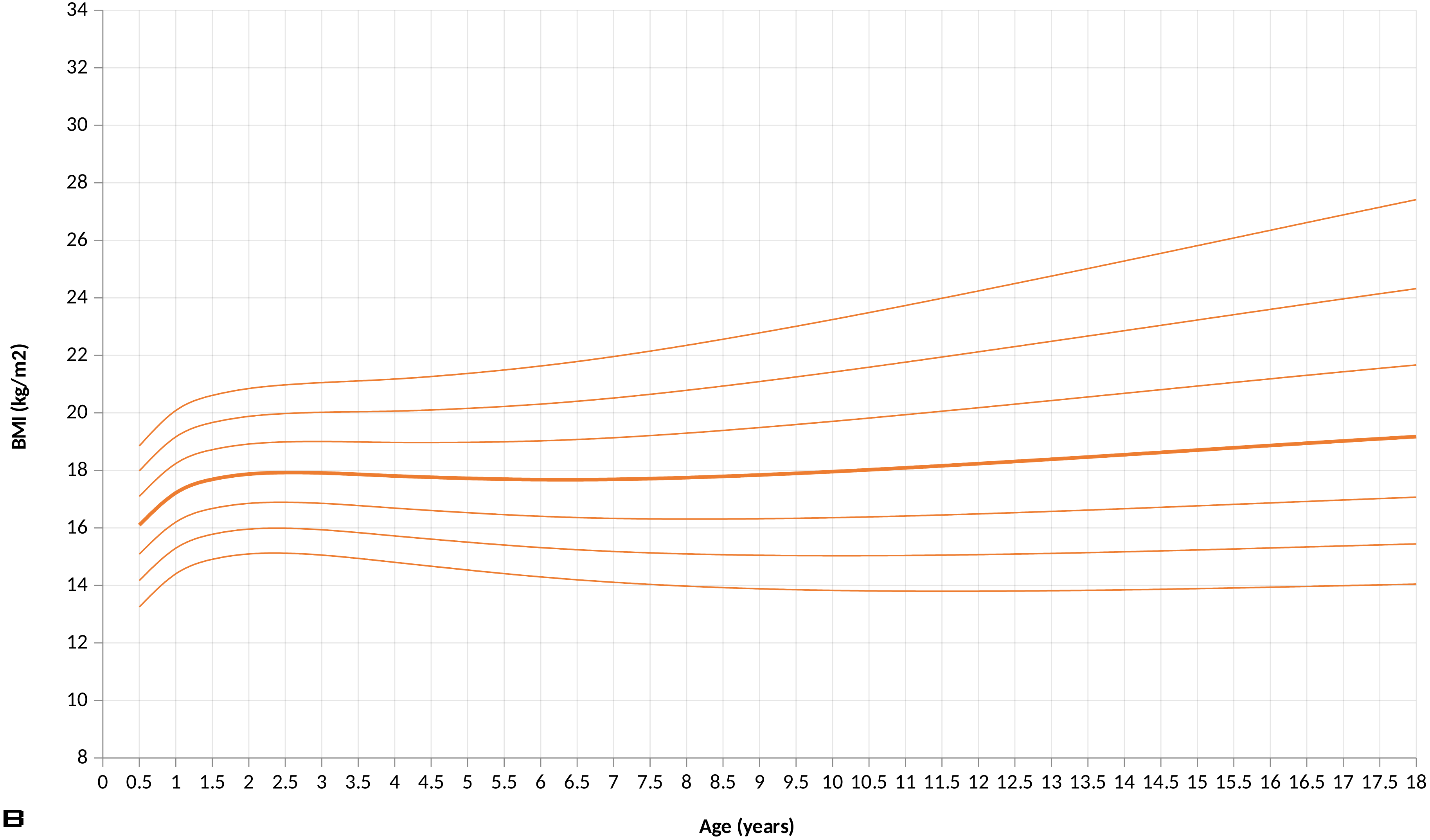
BMI (kg/m2)
34
32
30
28
26
24
22
20
18
16
14
12
10
8
0 0.5 1 1.5 2 2.5 3 3.5 4 4.5 5 5.5 6 6.5 7 7.5 8 8.5 9 9.5 10 10.5 11 11.5 12 12.5 13 13.5 14 14.5 15 15.5 16 16.5 17 17.5 18
Age (years)

B
